# Experience of use of dalbavancin for the treatment of unlicensed indications in a UK tertiary infectious diseases setting

**DOI:** 10.1007/s15010-025-02585-x

**Published:** 2025-06-23

**Authors:** Christopher A. Darlow, Joseph Parsons, Danielle Lucy, Ang Li, Libuse Ratcliffe, Stacy Todd, Nicholas Wong

**Affiliations:** 1 Antimicrobial Therapeutics and Pharmacodynamics, University of Liverpool, Liverpool, UK; 2 Tropical and Infectious Diseases Unit, Liverpool University Hospitals NHS Foundation Trust, Liverpool, UK; 3 Liverpool Clinical Laboratories, Liverpool University Hospitals NHS Foundation Trust, Liverpool, UK

**Keywords:** Dalbavancin, Infective endocarditis, Staphylococcus aureus, Bacteraemia, Boint and joint infection, Osteomyelitis, Skin and soft tissue infection, Outpatient antibiotic therapy, PWIDs, Off-licence, Lipoglycopeptides

## Abstract

**Background:**

Dalbavancin is a long-acting lipoglycopeptide with Gram-positive activity, licensed for the treatment of acute bacterial skin and skin-structure infections (ABSSSIs), although off-licence use is increasingly prevalent. We describe our experience in Liverpool of using dalbavancin for off-licence indications and as a risk-reduction strategy in patients at risk of premature hospital discharge.

**Methods:**

Patients receiving dalbavancin in the period 1/9/2020–30/4/2024 in Liverpool were identified. Data was extracted by review of patient notes. Primary outcomes were clinical success (resolution of infection without re-admission or further antibiotics) and 90-day mortality.

**Results:**

Ninety-five individual dalbavancin courses were identified. 24/95 were for licensed indications (i.e., ABSSSI without bacteraemia). Off-licence indications included bone and joint infections (BJIs) (30/95), infective endocarditis (IE) (13/95) and *Staphylococcus aureus* bacteraemia (SAB) (27/95). The clinical success rate and 90-day mortality for ABSSSI without bacteraemia were 91.67% and 4.17%, respectively. BJI without bacteraemia and SAB outcomes were similar (*p* > 0.999). However, IE had worse rates of clinical success (61.5%, *p* = 0.072) and 90-day mortality (30.8%, *p* = 0.042). 10/18 PWIDs who were prematurely discharged achieved clinical success; 17/18 were alive at 90 days.

**Conclusion:**

The data in this retrospective analysis adds to the growing body of evidence that dalbavancin is safe and effective for the treatment of BJIs and SABs. It also reinforces the uncertainty in the literature over the efficacy of use in IE. Additionally, these data demonstrate that dalbavancin may be used successfully as a risk mitigation strategy for PWIDs who may be prematurely discharged from an inpatient stay.

## Key points

These data adds to the growing body of evidence that dalbavancin is likely to be safe and effective for off-licence indications.

Risk mitigation for patients at risk of premature discharge with dalbavancin treatment is seemingly safe and effective.

## Introduction

Dalbavancin is a semi-synthetic lipoglycopeptide, derived from a teicoplanin-like molecule produced by the actinomycete *Nonomuraea* species [1]. Like the related glycopeptide group of antibiotics, it binds to D-alanyl-D-alanine residues in Gram-positive bacterial cells walls, preventing transpeptidation and transglycosylation, and leading to bacterial cell death [1, 2]. However, dalbavancin contains a lipophilic side chain that enhances antimicrobial potency by anchoring the drug to the bacterial cell wall compared to glycopeptides [3]. Additionally, dalbavancin has widespread tissue distribution and extensive protein-binding, leading it to have a unusually long half-life [4] allowing for a single 1500 mg intravenous infusion to achieve therapeutic levels for two weeks, and up to six weeks if another 1500 mg infusion is given one week later [5]. Like the glycopeptides, it has broad Gram-positive spectrum of activity, including to Methicillin-resistant *Staphylococcus aureus* (MRSA) and enterococci (although not to VanA-producing enterococci) [2, 3]. 

Dalbavancin was first approved in Europe and the USA in 2014 for the treatment of acute bacterial skin and skin structure infections (ABSSSI) based on trial evidence limited to that indication [1, 6]. Despite this relatively limited licence indication, dalbavancin is increasingly used for off-licence indications including bacteraemias [7–12], infective endocarditis (IE) [8, 9, 13–18] and bone and joint infections (BJIs) [19–23]. However, despite these available data, overall evidence remains non-definitive.

In Liverpool, we use dalbavancin for off-licence indications for two main reasons. First, as an alternative to daily Outpatient Antibiotic Therapy (OPAT), where logistical constraints prevent this. In the UK, dalbavancin is currently more expensive than once-daily OPAT with e.g. ceftriaxone or teicoplanin. However, it remains more cost-effective than an in-patient stay for the duration of treatment [24]. Secondly, we administer dalbavancin to mitigate harm for patients who would normally require inpatient intravenous antibiotic therapy but are unable to stay in hospital for behavioural reasons or are discharging against medical advice. We undertook a service evaluation to review our outcomes using dalbavancin in this context.

## Methodology

### Patient identification

All patients receiving dalbavancin in the period 1/9/2020–30/4/2024 at Liverpool University Hospitals NHS Foundation Trust (LUHFT) were identified from hospital pharmacy records. All administrations were recorded, as the drug was released on a named patient basis. Of these, all patients with medical records on the LUHFT electronic patient record (EPR) platform were included in the retrospective analysis.

### Data extraction

All patients had their EPR and digital prescription records manually reviewed, with data extracted regarding demographics, clinical details, antibiotic administration details (including dalbavancin and other antibiotics), microbiological data and outcome data. Primary outcomes measures including clinical success, defined by clinical resolution of infection without readmission or further course of antibiotics, and 90-day mortality. Any adverse events attributable to the dalbavancin administration were also recorded.

### Data analysis

Descriptive data for each parameter was collated and described using Microsoft Excel. Differences in outcomes were calculated for ABSSSI without bacteraemia (i.e. the licensed indication) vs. specific unlicensed indications using Fisher’s Exact Test.

### Patient consent statement

This retrospective analysis was conducted as part of a service evaluation approved by the Audit Committee of LUHFT. According to UK Health Research Authority regulations, service evaluations do not require specific research ethics approval.

## Results

**Fig. 1:**
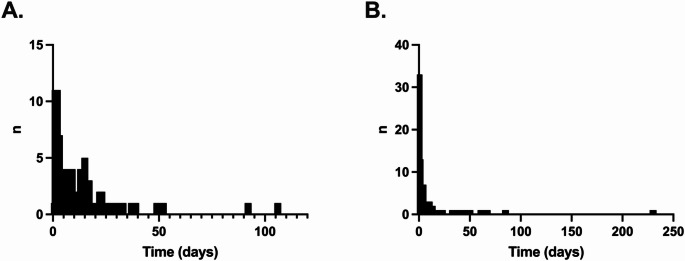
Frequency distributions of length-of-stay pre-first dose of dalbavancin (**A**) and post-first dose of dalbavancin (**B**) for patients given dalbavancin as an inpatient; excludes three patients given dalbavancin only as an outpatient

In the period 1/9/2020–30/4/2024, EPR-derived data was available for 89 individuals receiving 95 individual courses of dalbavancin. The demographics for these individuals and their episodes are shown in Table 1. A majority were male (66.3%), and a majority (55.79%) were (ex- or current) people who inject drugs (PWIDs). Significant numbers had complex housing needs post-discharge (40.0%), and behavioural issues complicating admission (43.15%). Median serum creatinine was 72 mmol/L, with a median estimated glomerular filtration rate (eGFR) of > 90mL/min/1.73m^2^. Only two individuals had an eGFR < 30 mL/min/1.73m^2^ necessitating a renal dose adjustment as per the dalbavancin SPC. The median length of stay (LoS) was 8.5 days before dalbavancin was given and 1-day post-dalbavancin administration. There were a small number of individuals with prolonged LoS post-dalbavancin (Fig. 1). These individuals had been given dalbavancin as a risk mitigation due to threatened unplanned discharge, but remained as an inpatient in the eventuality.

**Table 1:** Patient demographics. PWID = Person who inject drugs. Denominator is the total number of episodes requiring a dalbavancin course. Two individuals had two courses; one individual had five.

Characteristic	
Median Age (SD)	50 (15.7)
Males	63 (66.32%)
No fixed abode	18 (18.9%)
Hostel resident	20 (21.05%)
PWID (ex or current)	53 (55.79%)
Behavioural issues complicating admission	41 (43.15%)
Median Length of Stay (days)	
Pre-dalbavancin	8.5 (range 0–106)
Post-dalbavancin	1 (range 0–230)

### Indications and microbiology

The indications that dalbavancin were used for are noted in Table 2 with identified microbiological cause of the infection in Table 3. 24/95 were within the licensed indication (i.e. ABSSSI without bacteraemia). The main off-licence uses were BJIs (30/95, 31.6%), IE (13/95, 13.7%) and *S. aureus* (methicillin-susceptible or -resistant) bacteraemias (27/95, 28.4%). Other indications were heterogenous. 15/95 (15.79%) were associated with non-Staphylococcal bacteraemias. 7/95 (7.3%) were complex deep soft-tissue infections, such as pyomyositis and psoas abscesses. 6/95 (6.3%) were other infections including community-acquired pneumonia, empyema, intravascular device infection, line infection and mycotic aneurysm.

**Table 2:** Indications for dalbavancin therapy. Complex deep soft tissue infection includes myositis and deep-seated abscesses in soft tissue. ^1^licenced indication; ^2^ Includes three *Streptococcus agalactiae* and four *Streptococcus dysgalactiae*; ^3^Includes two *Enterococcus faecalis* bacteraemias with Gram-negatives, and one mixed culture of *E. faecalis*, Group B *Streptococcus* and MSSA. This latter culture was not included in the *S. aureus* bacteraemia analysis. ^4^ One *E. faecalis* bacteraemia and one *Streptococcus anginosus* group bacteraemia. The bacteraemia cases are categorised both as bacteraemias, but also as other indications where the bacteraemia is secondary to the identified source

Indications	Dalbavancin *n* = 95
ABSSSI	31 (32.6%)
*Without bacteraemia* ^1^	*24 (25.3%)*
*With bacteraemia*	*7(7.37%)*
Bone and joint infection (BJIs)–	
*Without bacteraemia*	19 (20.0%)
*With bacteraemia*	11 (11.58%)
Infective Endocarditis	13 (13.54%)
Bacteraemia	41 (43.15%)
MSSA	22
MRSA	5
β-haemolytic *Streptococcus* species^2^	7
Mixed^3^	5
Other Gram-positive^4^	2
Complex deep soft tissue infection	7 (7.37%)
Other	6 (6.32%)

**Table 3:** Identified microbiological cause of infection treated with dalbavancin. ^1^Includes 2 mixed bacteraemias with Gram-negatives;^2^Includes one mixed bacteraemia with *Gemella* and *Veillonella* species

Organism	Dalbavancin *n* = 95
No organism identified	52 (54.7%)
MSSA	23 (24.2%)
MRSA	5 (5.26%)
*Enterococcus faecalis* ^1^	3 (3.16%)
β-haemolytic streptococci	7 (7.37%)
Mixed	2 (2.11%)
*Streptococcus anginosus* group^2^	2 (2.11%)

A definitive causative organism was identified in 43/95 (45.3%) cases. *S. aureus* (either MRSA or MSSA) was the most frequent in 28/43 (65.11%) cases with an identified organism. In addition to those shown in Table 3, an *Escherichia coli* bacteraemia was also coincidentally present in a patient treated for ABSSSI, but this has not been included the data or further analysis.

### Antimicrobial regimens

All patients received an initial 1.5 g dalbavancin infusion, with the exception of two patients with an eGFR of < 30 mL/min/1.73m^2^, who received a 1 g dalbavancin infusion. 50/95 (52.6%) required a second dose one week later to achieve are required up to six weeks dalbavancin drug exposure. All except 10 received this as planned. Of the ten, seven failed to attend the follow-up appointment, two were re-admitted before second dose due, and one was transferred to another trust without further immediate follow-up.

### Outcomes

The clinical outcomes for ABSSSI with bacteraemia (as the licensed indication) and BJI without bacteraemia, *S. aureus* bacteraemia and IE are shown in Table 4, with a statistical comparison for illustrative purposes. BJI with bacteraemia were excluded from this analysis due to the multiple overlapping indications. Of these selected BJIs, five potentially required source control. Three had these controlled with surgery (one with metalwork that required removing); two were treated conservatively. Only one patient had metalwork involved with the infection. Both BJI and *S. aureus* bacteraemia had similar outcomes to the licensed indication (*p* > 0.999 for both outcome measures).

**Table 4:** Clinical outcomes for the most prevalent indications. *S. aureus* bacteraemias include MSSA and MRSA. *P* values calculated with Fisher’s Exact test in comparison to ABSSSI without bacteraemia

Indication	Clinical success *n* (%)	*P* Value	90 Day mortality *n* (%)	*P* value
All cases (*n* = 95)	80 (84.2%)	N/A	6 (6.32%)	N/A
ABSSSI without bacteraemia (*n* = 24)	22 (91.67%)	N/A	1 (4.17%)	N/A
Bone and joint infection without bacteraemia (*n* = 19)	17 (89.5%)	> 0.999	1 (5.26%)	> 0.999
*S. aureus* bacteraemia (*n* = 27)	24 (88.9%)	> 0.999	1 (3.7%)	> 0.999
Infective Endocarditis (*n* = 13)	8 (61.5%)	0.072	4 (30.8%)	0.042

However, IE had a lower clinical success rate (61.5% vs. 91.67%) and a higher mortality (30.8% vs. 4.17%), with *p* values of 0.072 and 0.042, respectively. These cases were high risk with 11/13 (84.6%) deemed as requiring surgery for valvular dysfunction secondary to the IE. Of these, only 2/11 (18.2%) actually had surgery, with the rest either refusing, non-compliant with treatment or deemed too high-risk for surgery. All IE treatment failures and mortality occurred in this latter group not receiving required surgery. 11/13 (84.6%) cases were native valve endocarditis. The two prosthetic valve endocarditis cases were the same individual who had two attempted and separate courses of dalbavancin 10 months apart. Surgery was required, but did not happen, for both episodes.

Of the seven patients who did not attend for a second dose, all had clinical success and were alive at 90 days. Eighteen of the dalbavancin courses were given as risk mitigation for patients who self-discharged (*n* = 14) or were evicted from the hospital for behavioural reasons (*n* = 4). All were PWIDs and 16/18 (88.9%) were homeless or lived in a hostel. Of these, 17 (94.4%) were alive by Day 90. However, 8/18 (44.4%) had clinical failure requiring further treatment or readmission. The indications for treatment of these 18 patients, delineated by clinical success, are detailed in Table 5. A further and separate group of 17 patients who were PWIDs had recorded behavioural issues that threatened premature discharge, but ultimately did not. Although their eventual discharge was planned, the dalbavancin was given either to mitigate harm in the case of unplanned discharge or to facilitate outpatient treatment without need for OPAT. 16/17 (94.1%) of these achieved clinical resolution of infection, and all were alive at 90 days.

No patient experienced any adverse events attributable to dalbavancin administration, save for clinical failure.

**Table 5:** Indications for use of dalbavancin in 18 patients who prematurely discharged, where dalbavancin was used as a risk mitigation strategy. ^1^Includes groin and psoas abscesses. ^2^*E. faecalis* bacteraemia with source not identified at point of discharge

Indication	Total	Clinical Failure
Bone and Joint Infection	6	1
Infective Endocarditis	4	3
ABSSSI	4	2
Soft tissue abscess^1^	2	2
Bacteraemia^2^	1	1

## Discussion

Our experience in Liverpool agrees with previous data suggesting efficacy for dalbavancin for a number of off-licence indications, particularly BJIs and *S. aureus* bacteraemia (SAB). In BJIs without bacteraemia, we achieved a 89.5% clinical success rate, comparable to previously published data collated by two recent reviews [19, 20]. Although this collective evidence, along with our own, is reassuring, this is all retrospective data. Prospective trials for the use of dalbavancin for the treatment of BJIs are needed to definitively determine efficacy of this treatment.

There is a smaller but increasing body of published data for the efficacy of dalbavancin for the treatment of SAB. One US large multicentre retrospective study of dalbavancin use for the treatment of bacteraemias (*n* = 115, of which 83/115 [72%] were SAB) had an overall clinical success rate of 87.8% for Gram-positive bacteraemias, although they didn’t delineate outcome data by causative organism [7]. Further smaller retrospective studies examining SAB in particular have similar comparable treatment rates [10, 11]. A post-hoc analysis of the relatively small number of SABs within the original DISCOVER trials, revealed a similar success rate, and apparent superiority to vancomycin/linezolid, although the numbers were too small to perform a statistical analysis [12]. However, a Phase 2b trial examining the efficacy of dalbavancin for the treatment of SAB [25] has been completed. The data has yet to be published in a peer-reviewed format, but the released data associated with the clinicaltrials.gov entry (NCT04775953) indicate non-inferiority to standard-of-care treatment. Our clinical success rates with dalbavancin for SAB (88.9%) is entirely consistent with these available data.

Our clinical success rates for IE (61.5%) were poorer than for other examined indications. This may represent a true difference in efficacy, but likely reflects a selection bias of our patients. The IE patients receiving dalbavancin in Liverpool were, in general, high risk IE cases and all treatment failures and mortality occurred in patients requiring surgery, but did not receive it. Although the statistical significance was around the standard threshold of significance, we made this comparison with ABSSSI for illustrative purposes only. To make a true inference of the efficacy of dalbavancin for IE, a comparison with matched non-dalbavancin treated IE patients is required. Whilst we could not collect data for this within the ethical approval limits of this study, clinical success and mortality were better (84.4% and 8.5%, respectively) for patients receiving standard-of-care treatment for IE in a large recent multi-centre retrospective study [26]. Reported rates of success from a recent systematic review of the available data and an additional dataset published since the systematic review [13, 14] reveal that reported rates of success varies significantly from 50 to > 90% for IE treated with dalbavancin. All published datasets are heterogenous in dalbavancin dosing and clinical context, small in number, and subject to a general high degree of bias due to retrospective non-randomised analysis. Accordingly, the safety and efficacy of dalbavancin in the treatment of IE is uncertain, and our data is in accordance with that. Consequently, further data is required to gain more certainty about the efficacy of dalbavancin for the treatment of IE. We agree with Leanza et al. [13] that whilst dalbavancin may well be a promising treatment of IE, currently it should be used with caution, until less biased data is available, ideally with prospective randomised controlled trials.

Our study joins other retrospective analyses of dalbavancin for off-licence indications [27–36], including two UK cohorts [30, 34]. All of these cohorts are relatively small and, individually, give limited confidence in the use of dalbavancin in off-licence indications. All are also biased by being retrospective analyses of real-world use– the apparent success rates may be due to careful patient selection by clinicians, rather than effect of the drug.

What our cohort adds to these is experience of use of dalbavancin as a risk mitigation strategy for vulnerable patients who, for one reason or another, fail to stay in hospital for as long as required for conventional inpatient antimicrobial therapy. A majority of our patients were PWIDs, usually complicated by complex housing situations (e.g. homelessness or living in hostels). PWIDs are at risk of infections dalbavancin may be effective against (e.g. SAB, IE, BJIs, ABSSSIs) [37], are at increased risk of discharge against medical advice [38, 39], and OPAT services are considered suboptimal for this group [40]. In this context, dalbavancin has particular benefits. It ensures adequate drug exposure comparable to intravenous therapy, even if the patient self-discharges or fails to attend follow-up. It also relieves the need to use an in-dwelling long-term venous catheter to facilitate OPAT, which have a higher risk of developing line infection in this cohort [40]. As such, dalbavancin is an optimal choice over alternatives (no treatment or sub-optimal oral antimicrobials) if the patient prematurely discharges, and a more reliable option for sequential therapy instead of OPAT.

In our analysis, all of the 18 patients who prematurely discharged were PWIDs. Although the clinical failure rate was higher than ideal (8/18, 44.4%), 90-day mortality was low (1/18, 5.5%), and the overall outcomes likely to be better than the alternatives (no treatment or sub-optimal oral antimicrobials). In conjunction with the 17 PWIDs who had behavioural issues threatening premature discharge, the overall outcome (26/35 clinical success and 34/35 90-day survival) was acceptable for a high-risk cohort for whom the alternative would likely have led to worse outcomes. As such, this experience leads us to conclude that dalbavancin use as a risk mitigation strategy is a likely efficacious and safe treatment choice. However, further data is needed, ideally prospectively collected, with non-dalbavancin treated controls to provide stronger evidence.

There are limitations to this data. Like much of the aforementioned published data on dalbavancin use for unlicensed indications, it is retrospective in nature, without a direct comparator using standard-of-care treatment.

## Conclusion

Our retrospective cohort analysis has added to the body of existing evidence (described above) that use of dalbavancin for the treatment of BJI and SAB is likely efficacious and safe, whilst raising caution in its use for IE. Further, the study has provided evidence of safe and efficacious use as risk mitigation strategy for patients threatening or actioning premature discharge.

## Data Availability

Data cannot be shared openly for reasons of study participant privacy and information governance restrictions. Data can be shared, with the appropriate approvals and restrictions, by contacting the corresponding author.
